# The Effect of Chronic Wasting Disease on Resident Deer Hunting Permit Demand in Wisconsin

**DOI:** 10.3390/ani9121096

**Published:** 2019-12-07

**Authors:** Dane Erickson, Carson Reeling, John G. Lee

**Affiliations:** Department of Agricultural Economics, Purdue University, West Lafayette, IN 47907, USA; ericks21@purdue.edu (D.E.); jlee1@purdue.edu (J.G.L.)

**Keywords:** chronic wasting disease, deer hunting, permit demand, Wisconsin

## Abstract

**Simple Summary:**

Chronic wasting disease is a fatal neurodegenerative disease that affects cervids across the world. While chronic wasting disease is not known to be transmissible to humans, there is increasing concern about chronic wasting disease risks, particularly among hunters who may contact and consume infected animals. We examine demand for resident deer hunting permits in Wisconsin from 1966 to 2015 to quantify the losses to deer hunters following the discovery of chronic wasting disease (CWD) in 2002. We estimate that permit demand decreased by >5% following the discovery of the disease. Consumer surplus—or the dollar value of permits to Wisconsin deer hunters—declined by $96 million between 2002 and 2015, while permit revenues decreased by nearly $17 million. The effects of the disease’s presence slowly diminish over time. This is because total demand for permits would have declined over this period even in the absence of the disease. These findings highlight the need for joint management of both chronic wasting disease risks and hunter participation; intuitively, the economic value of protecting a resource declines with the number of use people that use it.

**Abstract:**

Chronic wasting disease (CWD) has had a negative impact on deer license demand in Wisconsin since it was first found in the state in 2002. Prior work evaluates the effect of CWD on hunting permit sales, but only in the period immediately after the disease was discovered. We use data on hunting permit sales, permit price, and other demand shifters to estimate a model of deer permit demand for the period 1966–2015. We use the estimated model to quantify the effect of CWD on (1) hunter demand for deer permits; (2) hunter surplus from hunting; and (3) lost hunting permit revenues. Hunter participation declined by 5.4% after CWD was detected in 2002. Hunter surplus decreased by $96 million over this period, while permit revenues declined by nearly $17 million. The effect of CWD was greater on demand for firearm permits than for archery permits. We also find that the effects of CWD diminish over time in absolute terms. This is because permit demand would have started to decline in 2008 even in the absence of CWD. This finding implies efforts to control CWD and efforts at hunter recruitment are economic complements and should be pursued jointly to maximize hunter welfare.

## 1. Introduction

**Figure 1:**
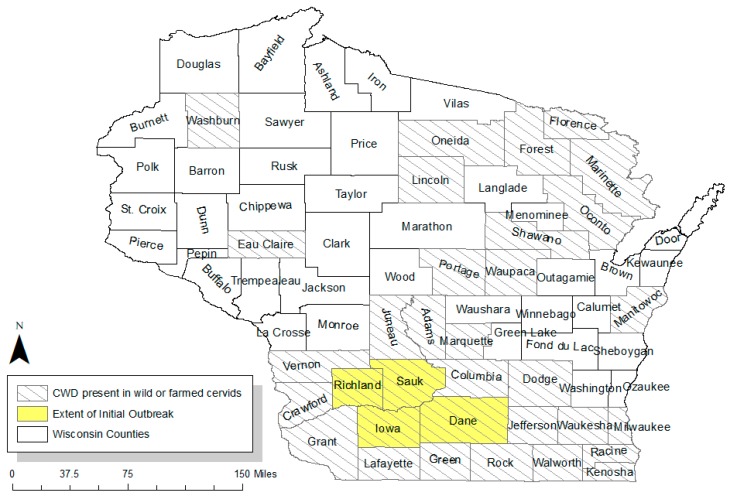
Chronic wasting disease (CWD) prevalence in Wisconsin (source: adapted from [5])**.**

Wisconsin has a strong tradition of white-tailed deer hunting, with regulated hunting first taking place over 150 years ago [1]. In early 2002, all of this was thought to be threatened by chronic wasting disease (CWD), a highly contagious neurodegenerative disease with a 100% fatality rate [2]. The agent of disease spread in the infected animal, prions, are shed when the animal excretes bodily fluids or dies and may remain infectious while in the soil for years, making the disease difficult to remove from an area [3]. The persistence of this disease and the rapid rate at which it can spread made containment a top priority for Wisconsin when it was first detected in three deer. Wisconsin was unique among other states in the panic that ensued as a result of CWD presence; the state government treated the disease as if it were a fire to be stamped out [4]. The Wisconsin government and its state game agencies raced to pass laws to contain the disease and implemented aggressive regulatory changes. Management activities included eliminating the deer population within the 411 square mile epicenter around the location of the initial discovery, which includes most of Richland, Sauk, and Iowa Counties, and the western part of Dane County (Figure 1). Other regulatory changes included liberalizing deer hunting quotas statewide to reduce overall deer population and implementing regulations against practices that encourage deer to congregate in large numbers (e.g., banning recreational feeding [4]).

CWD is not known to be transmissible to humans [6]. Yet, there is growing concern among wildlife managers, health officials, and deer hunters about the risks associated with consuming infected deer meat or handling infected carcasses [7]. Of particular concern is the effect of CWD status on deer hunting permit sales, particularly against the backdrop of declining hunter participation rates nationwide [8]. Hunting participation rates started to level off prior to the detection of CWD in Wisconsin in 2002 and began to decline thereafter. The losses to hunters and to Wisconsin’s hunting industry directly following the discovery of CWD in 2002 were immense. Wisconsin’s hunters spent a total of $553 million on deer hunting in 2001. The presence of CWD caused a loss of $55 million in annual revenue for the state’s hunting industry in 2002 alone [9]. These losses are consistent with experience in other areas following CWD outbreaks (e.g., Colorado), which experienced declining consumer demand for game meat, increased regulations for farmed cervid producers, and reduced hunting participation—along with lower revenues for both the state and businesses located in areas dependent on hunting [10].

The continued management of CWD requires understanding how hunters respond to an outbreak. Prior work examines how hunters may change hunting site choices following an outbreak (e.g., [11,12]). We study this response by examining changes in hunter demand for deer hunting permits. Demand for deer permits is an important metric for disease management. Indeed, permit sales are an important source of revenue for wildlife management agencies; 80% of the Wisconsin Department of Natural Resources (DNR) fish and wildlife account revenue comes from hunting and fishing license revenues [13]. Hunters can play a major role in population management to control infectious contacts. Finally, demand for permits can reveal measures of the economic loss hunters experience in infected states.

Prior work estimates the effects of CWD outbreaks on permit demand. Bishop [9] uses US Fish and Wildlife Data on total hunting expenditures (adjusted to approximate expenditures on deer hunting) to estimate the impact of CWD in Wisconsin; he estimates losses of up to $66 million statewide in the year 2003. Seidl et al. [10] use a similar approach to estimate losses due to CWD to the Colorado tourism, hunting, and farmed cervid industries. These prior studies are limited in that they examine losses only in the immediate aftermath of outbreaks. The level of panic with which Wisconsin wildlife agencies treated CWD likely served as a psychological factor, reducing deer permit demand during the 2002 hunting season [8], yet it is unlikely these losses persist through time. We use data on Wisconsin deer permit sales from 1966 to 2015 to study how Wisconsin hunters have responded to CWD over a longer period than previous research. Specifically, we estimate a model of hunter demand for deer permits following Loomis et al. [14] to quantify the changes in resident hunting permit demand, permit revenues, and hunter surplus following the 2002 CWD outbreak. We find initial decreases in demand due to CWD consistent with prior estimates [9], although the effect of CWD on permit demand diminishes over time. We estimate CWD reduced hunter surplus and hunting permit revenues by $96 million and $17 million, respectively, between 2002 and 2015. We also predict that permit demand in Wisconsin would have decreased starting in 2008 for structural reasons even in the absence of CWD. This finding implies that the economic importance of managing CWD is diminishing over time with hunter participation; managing the disease should be seen as a complement to other efforts aimed at improving hunter participation.

## 2. Materials and Methods

We collected data from several sources. Table 1 provides summary statistics for key variables used in our analysis.

The Wisconsin DNR provided us with data describing the quantity of deer permits sold statewide each year from 1966 to 2015 [15]. We focus on estimating the effect of CWD on statewide demand for permits for several reasons. First, permit demand data were not available at a finer spatial scale (e.g., county or deer management unit). Second, deer permit demand may depend on individuals’ imperfect information about the extent of the outbreak. Hunters may not know where the next positive deer would show up; hence, we would expect CWD to have effects on demand beyond the region of the initial outbreak. (Indeed, Figure 1 shows that the extent of the CWD outbreak has expanded since 2002.) Third, DNR changes factors like management unit boundaries and testing incentives yearly, complicating demand estimation at these smaller scales.

The deer season in Wisconsin is divided into separate archery and firearm seasons. Each type of season requires a separate permit, and we have permit sales data for each type. Both Wisconsin residents and non-residents are eligible to purchase deer permits, although nonresident permits tend to be considerably more expensive. We restrict our analysis to demand for resident permits for two primary reasons. First, nonresidents are likely to respond to CWD risks differently than residents. In particular, we anticipate nonresidents are more likely to substitute trips to neighboring states after an outbreak. We cannot capture this type of substitution behavior in our model (described later). Second, nonresident sales make up <4% of all Wisconsin license, permit, tag, and stamp sales on average; we therefore do not expect that focusing on resident demand will qualitatively affect our results. Our permit sales data is not separated into resident and non-resident sales. However, we were able to collect data on the share of all hunting permits for all regulated species in Wisconsin sold to residents and nonresidents for each year of our data [16,17]. We therefore estimate resident deer permit sales as the total permit sales times the share of all permits sold to Wisconsin residents.

**Figure 2:**
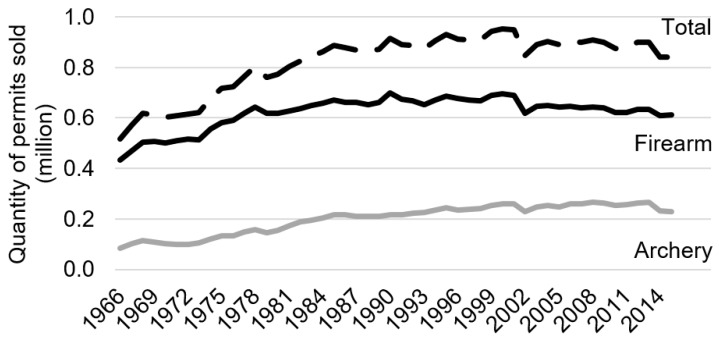
Wisconsin Deer Permit Demand, 1966–2015.

Figure 2 shows hunting permits purchased between 1966 and 2015. Wisconsin hunter demand for deer permits generally grew between the years of 1966 and 2000, with total permit demand reaching a peak of 952,711 in the year 2000. Permit demand plummeted by 102,710 permits after the discovery of CWD in 2002. This drop accounts for over 10% of the previous year’s permit sales. Total demand appears to have partially recovered in the years following the outbreak, although Figure 2 reveals that this recovery is due almost entirely to demand for archery permits, which continued to grow at nearly a constant rate after the outbreak. In contrast, the shock to firearm permit demand—by far the most popular form of deer hunting in Wisconsin—appears to have been more permanent; demand partially recovered after 2002 but continued to decline thereafter.

Aggregate demand for any good is typically assumed to be a function of prices, income, population, and other factors that affect consumers’ tastes and preferences. We collected resident permit price data from the Wisconsin DNR [1]. The price of firearm and archery permits is the same every year except for the period between 1973 and 1990, although the difference in price is less than $2 in nominal terms over this period. Permit prices rarely change from year to year in nominal terms; indeed, the unit price of permits changed only nine times over the fifty-year period covered by our data. Given this lack of variation and the long time period encompassing many changes in macroeconomic conditions, we deflate permit prices to 2012 dollars using the consumer price index [18]. We take data on Wisconsin mean per capita income from the U.S. Regional Economic Analysis Project [19], which compiles regional-level income estimates from the Bureau of Economic Analysis into state-level mean income estimates. Like permit prices, income is deflated to 2012 dollars. Ideally, we would use data on mean hunter income rather than income data for the population as a whole. However, we are not aware of any comprehensive source for data on hunter income. The US Fish & Wildlife Service (US FWS) publishes estimates of these data at the state level, but only for a few of the years covered by our study [20]. Fortunately, we find that the distribution of hunter income is statistically similar to income for the population as a whole (see the online supplemental material). State-level population estimates for Wisconsin come from the U.S. Census Bureau [21]. Finally, we capture changes in hunter tastes and preferences using a time trend and a binary variable indicating the presence of CWD in Wisconsin.

We use our data to estimate a demand function for deer hunting permits in Wisconsin from 1966 to 2015. Specifically, we model the natural logarithm of licensed deer hunters in Wisconsin as a function of permit price, income, a quadratic time trend, and the presence of CWD through a binary variable. Formally, the demand function is

ln(*q_t_*) = *α* + *βp_t_* + **γ**′**X***_t_* + *δ*_1_*CWD_t_* + *θ*_1_*t* + *θ*_2_*t*^2^ + *u_t_* (1)

where *q_t_* is the quantity of deer permit sales in Wisconsin in year *t* and **X***_t_* is a column vector of demand shifters (including state mean income and population). The variable *t* is the year (1966–2015), which captures changes in unobserved structural components of demand (e.g., population-level changes in preferences for hunting). *CWD_t_* is a binary variable equal to one if CWD is present in Wisconsin in year *t*, implying a shift in the demand curve in response to a CWD outbreak. Finally, *u_t_* is a random shock. The Greek characters are parameters to be estimated, and the prime symbol (′) denotes a matrix transpose.

We use the general specification in (1) to estimate several different demand functions. The first, which we refer to as “Model 1,” is the aggregate demand for both archery and firearm deer permits. Quantity demanded, *q_t_*, is the sum of firearm and archery permits sold to Wisconsin residents in each year. We set price in a given year equal to the weighted average of real firearm and archery permit prices, where the weights equal the proportion of each type of permit sold. Model 2 is the same as Model 1 except we include US Census estimates of Wisconsin state population as a demand shifter. Models 3 and 4 estimate separate demand functions for resident firearm and archery permits, respectively.

## 3. Results

Table 2 shows ordinary least squares parameter estimates for each model, with standard errors corrected for heteroscedasticity detected using a Breusch–Pagan test. (Data and estimation code are available in the online supplementary materials). Consider first the estimates for Model 1. All parameter estimates are statistically significant at the 1% level or better. The estimated parameter for permit price is negative, as is expected, and implies a marginal increase in price decreases permit demand by 0.7%. Likewise, we find that demand is decreasing in income—a $10,000 increase in mean per capita income decreases permit demand by 11%, implying deer hunting opportunities are an inferior good. This finding is consistent with prior work [22,23].

The effect of CWD on permit demand is negative and large in magnitude. Let

(2) $${\hat{q}}_{t}^{k}={e^{\hat{\alpha }+\hat{\beta }p_{t}+{\hat{\gamma }}^{\prime }X_{t}+{\hat{\delta }}_{1}CWD_{t}+{\hat{\theta }}_{1}t+{\hat{\theta }}_{2}t^{2}}|}_{CWD_{t}=k},\;k=0,\;1,$$

where a carat (^) denotes an estimate. Then the proportional change in baseline demand ($q_{t}^{0}$) due to CWD in Models 1−4 is

(3) $$\frac{{\hat{q}}_{t}^{1}-{\hat{q}}_{t}^{0}}{{\hat{q}}_{t}^{0}}=e^{{\hat{\delta }}_{1}}-1.$$

**Figure 3:**
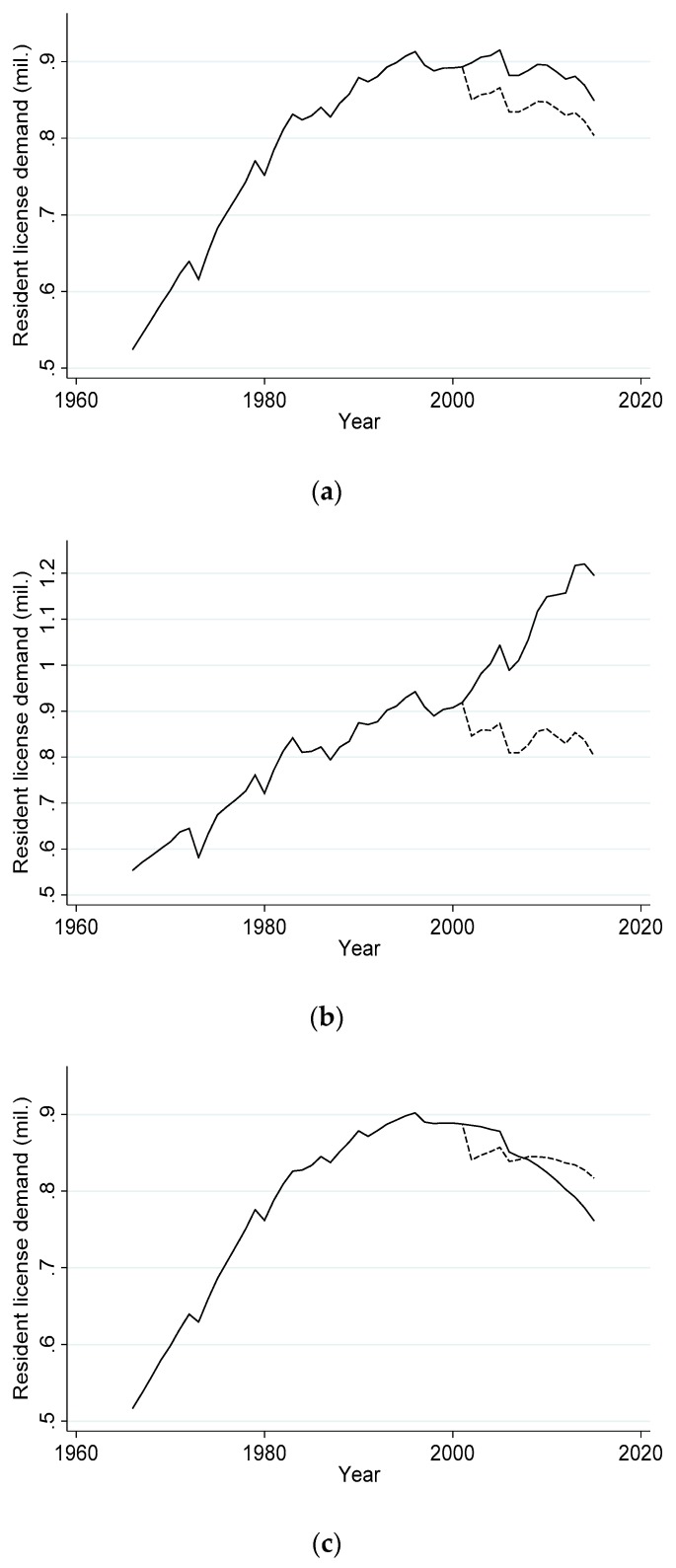
Estimated resident deer hunting license demand following the 2002 CWD outbreak (dashed line) and in the absence of CWD (solid line) for (**a**) Model 1, (**b**) Model 5 and (**c**) Model 6.

Given the parameter estimates in Table 2, Model 1 predicts that demand for deer permits decreased by 5.4% due to the outbreak. Figure 3a shows the effect of CWD on permit demand graphically. The dotted line shows estimated demand in the presence of CWD. The solid line shows predicted demand if no outbreak were to occur.

Next, consider Model 2. Neither the signs nor the magnitudes of the estimated parameters change significantly relative to Model 1, except the parameter for income becomes statistically insignificant. Model 2 predicts a smaller effect of CWD on permit demand; permit sales decrease by 4.2% after the outbreak.

Estimates for Models 3 and 4 reveal that the negative effects of CWD are borne exclusively by firearm hunters. Specifically, Model 3 predicts CWD caused a decrease in firearm permit demand of 5.9%. In contrast, Model 4 predicts CWD caused no statistically significant decrease in archery permit demand. This is consistent with the data shown in Figure 2, which revealed no noticeable effect of CWD on the growth of demand for archery permits.

Note from (3) that our demand model implies the loss in permit sales due to CWD is a constant proportion of baseline permit demand. Results from Models 1–4 show that total permit demand would have peaked between 2008 and 2011 even in the absence of CWD (Figure 3a). This implies the effect of CWD on permit demand is diminishing in absolute terms over time. We check the robustness of this finding by comparing the estimated effect of time on permit demand implied by (1) with alternative demand function specifications. Model 5 specifies demand as

ln(*q_t_*) = *α* + *βp_t_* + **γ**′**X***_t_* + θ_1_*t* + *δ*_1_CWD_t_ + *δ*_2_*CWD_t_* ⋅ *t* + *u_t_* (4)

such that the proportional change in permit demand, $\left({\hat{q}}_{t}^{1}-{\hat{q}}_{t}^{0}\right)/{\hat{q}}_{t}^{0}=e^{{\hat{\delta }}_{1}+{\hat{\delta }}_{2}t}-1$, varies with time. We also estimate Model 6, which specifies demand as

ln(*q_t_*) = *α* + *βp_t_* + **γ**′**X***_t_* + *θ*_1_*t* + *θ*_2_*t*^2^ + *δ*_1_*CWD_t_* + *δ*_2_*CWD_t_* ⋅ *t*^2^ + *u_t_* (5)

such that the proportional change in permit demand due to CWD is $\left({\hat{q}}_{t}^{1}-{\hat{q}}_{t}^{0}\right)/{\hat{q}}_{t}^{0}=e^{{\hat{\delta }}_{1}+{\hat{\delta }}_{2}t^{2}}-1$.

Table 2 shows estimates for Models 5 and 6. Estimated parameters are all significant at the 10% level or better for these alternative models except for income in Model 6. Yet the estimated parameters imply counterintuitive behavior on the part of applicants in the absence of CWD, as shown in Figure 3b–c. The linear trend term in Model 5 implies resident permit demand would have continued to increase at a constant rate in the absence of CWD. This is unlikely given that demand appears to have flattened out in the late 1990s (Figure 2). The overall fit of this model is also inferior to that of Model 1, albeit only slightly (R^2^ is 0.9253 for Model 5 compared with 0.9638 for Model 1). We therefore discard Model 5 in favor of Model 1. While the overall fit of Model 6 is superior to Model 1 (R^2^ is 0.9668), the estimated parameters for Model 6 predict that demand for permits in the presence of CWD would be greater than demand in the absence of CWD starting in 2008 (Figure 3c). This finding is unlikely, and so we discard Model 6 in favor of Model 1 as well.

Model 1 reveals permit sales declined by ~48,000 permits per year on average after 2002. In real terms, expenditures on permits declined by >$16.6 million in real terms due to CWD over this period. Hunter surplus, or the dollar value of permits to hunters in excess of permit cost, is given by 

(6) $$S_{t}=\int_{p_{t}}^{\infty }e^{\hat{\alpha }+\hat{\beta }p+\hat{\gamma }X_{t}+{\hat{\delta }}_{1}CWD_{t}+{\hat{\theta }}_{1}t+{\hat{\theta }}_{2}t^{2}}dp=-\frac{1}{\beta }{\hat{q}}_{t}^{0}.$$

**Figure 4:**
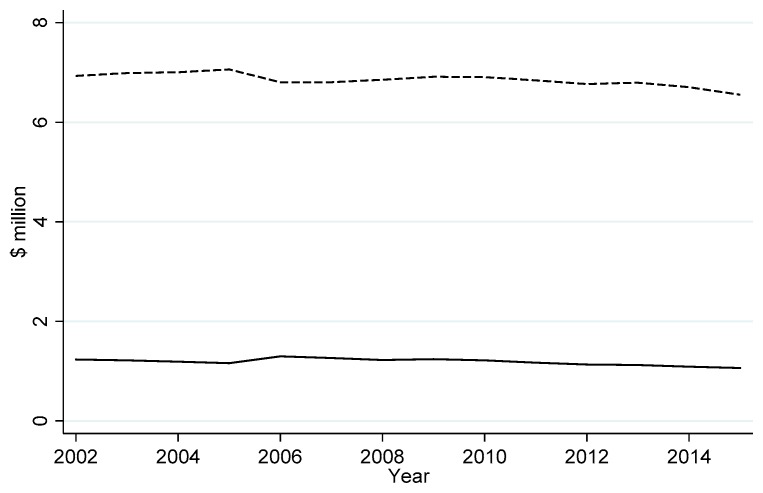
Estimated real foregone resident deer hunting permit expenditures (solid line) and lost resident deer hunter surplus (dashed line) after the chronic wasting disease outbreak in Wisconsin, 2002–2015.

Model 1 predicts the loss in surplus from CWD is nearly $96 million from 2002–2015. Figure 4 shows estimated lost revenues and loss surplus due to CWD over the outbreak period. 

## 4. Discussion

The effects of CWD on permit demand are significant but diminish over time. Figure 4 summarizes these effects; note that losses to both hunter surplus and permit revenues from CWD decrease over time (albeit slowly). Mathematically, the loss in hunter surplus from CWD is

(7) $$\Delta S_{t}=\frac{1}{\left|\hat{\beta }\right|}\left({\hat{q}}_{t}^{1}-{\hat{q}}_{t}^{0}\right)=\frac{1}{\left|\hat{\beta }\right|}\left(e^{\hat{\delta }}-1\right){\hat{q}}_{t}^{0}$$

which changes with time according to

(8) $$\frac{\partial \Delta S_{t}}{\partial t}=\frac{1}{\left|\hat{\beta }\right|}\left(e^{\hat{\delta }}-1\right)\left({\hat{\theta }}_{1}+2{\hat{\theta }}_{2}t\right)q_{t}^{0}.$$

**Figure 5:**
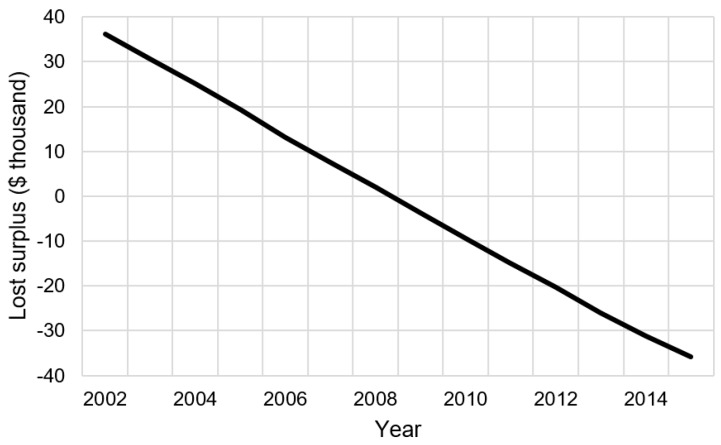
The effect of time on the losses in hunter surplus due to CWD, 2002–2015.

Figure 5 shows this effect graphically. Note that, immediately following the outbreak, CWD diminishes total hunter surplus over time (i.e., the loss in surplus is greater than zero). After 2008, the loss in surplus diminishes over time (i.e., the loss in surplus is less than zero).

These effects result from secular changes in demand for resident hunting permits over time. Aggregate permit demand peaked in 2008 and decreased thereafter (Figure 3a). Since the proportional loss in demand due to CWD is constant over time, the loss in permit demand decreases in absolute terms over time.

**Figure 6:**
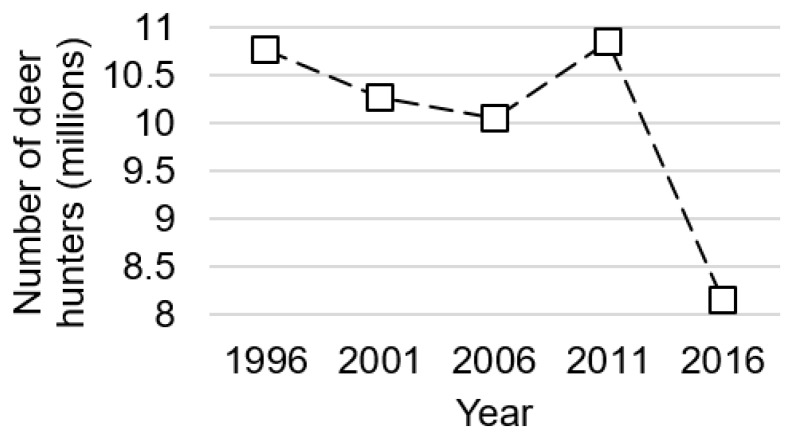
Number of US deer hunters, 1996 to 2016 (source: [20]).

**Figure 7:**
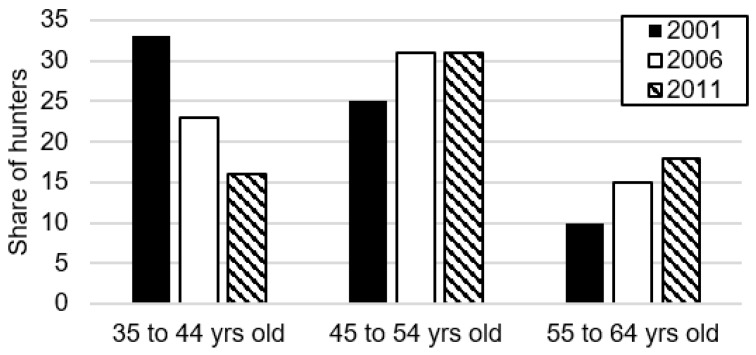
Percent share of Wisconsin hunters by age group, 2001–2011 (source: [20]).

Our model does not provide insight into what is driving the secular decrease in permit demand Wisconsin has observed since 2008, although the decline in Wisconsin matches declines observed nationwide; Figure 6 shows the estimated number of US deer hunters has declined from 10.8 million to 8.1 million between 1996 and 2016. Prior work suggests that an aging population of deer hunters may decrease permit demand, as older people are less likely to hunt [24,25,26,27]. Indeed, Figure 7 shows hunters are getting older in Wisconsin; the share of hunters between 35 and 44 years of age has steadily declined between 2001 and 2011, while the share of hunters between 55 and 64 years of age has steadily increased. Yet more recent work finds hunter participation is largely invariant to age until the hunter becomes ~65 years old; instead, the secular decrease in demand is more likely due to “cohort effects”, or differences in tastes and preferences for hunting across generations [28]. In particular, “Baby Boomers” (those born in the post-war period before the mid-1960s) have a stronger tradition of hunting relative to those born after 1980. As the Baby Boomers age out of hunting, they are less likely to be replaced at the same rate by younger hunters. What is clear from our model is that these secular trends will continue to cause permit demand to decline over time, all else equal. As permit demand decreases, so too will the benefits from managing CWD.

This finding has important implications for wildlife managers. In particular, our results suggest that management activities that reduce CWD prevalence and activities aimed at recruiting new hunters (i.e., activities that slow or reverse the secular decline in demand for deer permits) are economic complements: increasing the recruitment of new hunters increases the marginal benefits from management activities that reduce CWD prevalence. Our analysis is silent on how to balance CWD management activities with hunter recruitment. Yet our findings imply that CWD management strategies should be formed in tandem with other activities geared towards increasing participation in deer hunting. Intuitively, the economic value of protecting the deer herd from CWD declines with the number of people interested in hunting deer.

## 5. Conclusions

We estimate demand for resident deer hunting permits in Wisconsin following the 2002 CWD outbreak. We find that CWD decreased permit demand by 5.4% following the outbreak, resulting in a loss of $96 million in hunter surplus and nearly $17 million in permit sales revenues between 2002 and 2015. The effects of CWD on demand decrease over time with the population of deer hunters, implying efforts to mitigate CWD risks are economic complements to hunter recruitment.

Our findings are subject to some caveats. We focus exclusively on resident permit demand. The value of hunting opportunities is likely different for nonresidents than for residents. We do not know whether this value—measured by consumer surplus—is greater than or less than residents’ value ex ante. However, the effect of excluding nonresident demand on our results is likely to be small given that ~4% of total permits are sold to nonresident hunters. Our analysis also only captures a single aspect of the value of CWD management (i.e., lost surplus to deer hunters). We acknowledge that there are other benefits from reducing CWD incidence that we do not capture. These may include general equilibrium effects estimated by prior work [9] (e.g., more deer hunters implies more spending at sporting goods stores), the disutility experienced by non-hunters concerned about the spread of CWD among the deer herd (which may be significant [29]), option values for future hunting activities, the value of deer in supporting populations of other economically important species and ecosystems, and even the value of averted health risks to humans from infected deer.

Our results raise questions for future research. In particular, we find that demand for permits by archery hunters is invariant to CWD presence. There are several possible explanations for this observation, including a strong tradition of bow hunting in the state and technological improvements in bow hunting equipment. For example, crossbows became legal equipment for all hunters in 2013, and demand for permits among crossbow hunters is growing [30]. Future research investigating differences in disease risk preferences across different hunter types would help wildlife officials prioritize disease management activities.

## Figures and Tables

**Table 1:** Summary Statistics.

Variable	Obs.	Mean	Std. Dev.	Min.	Max.
*Dependent variables*					
Quantity of resident archery permits sold	50	192,783.7	54,235.1	83,099	253,186
Quantity of resident firearm permits sold	50	600,415.1	61,223.3	421,880	681,063
*Explanatory variables*					
Resident firearm permit price ($ real)	50	12.13	1.42	9.99	16.33
Resident archery permit price ($ real)	50	11.77	1.44	8.61	15.38
Wisconsin mean per-capita income ($10,000)	50	3.14	0.90	1.68	4.69
Time trend	50	1990.5	14.6	1966	2015
Chronic wasting disease present = 1	50	0.28	0.45	0	1
Wisconsin population (million)	50	5.02	0.47	4.27	5.76

**Table 2:** Estimated Demand Equations for Wisconsin Resident Deer Hunting Permits ^1^.

Parameter ^2^	Variable	Model 1	Model 2	Model 3	Model 4	Model 5	Model 6
*α*	Constant	−1642.6 *** (139.12)	−1609.2 *** (148.46)	−1434.5 *** (123.86)	−2691.1 *** (286.15)	−47.241 *** (6.6160)	−2143.8 *** (339.83)
*β*	Real permit price ($2012)	−0.0070 *** (0.0022)	−0.0073 *** (0.0022)	−0.0081 *** (0.0020)	−0.0006 (0.0034)	−0.0127 *** (0.0030)	−0.0052 * (0.0027)
*γ*_1_	Mean annual WI income in tens of thousands ($2012)	−0.1130 *** (0.0362)	−0.0614 (0.0488)	−0.0875 ** (0.0323)	−0.2432 *** (0.0806)	−0.3225 *** (0.0506)	−0.0286 (0.0648)
*γ*_2_	WI population in millions	−	−0.1667 * (0.0971)	−	−	−	−
*δ*_1_	CWD = 1 if present, = 0 otherwise	−0.0554 *** (0.0185)	−0.0429 * (0.0224)	−0.0607 *** (0.0170)	−0.0322 (0.0334)	44.192 *** (5.0574)	−9.4960 * (5.4128)
*δ*_2_ (model 5)	CWD × time trend	−	−	−	−	−0.0221 *** (0.0025)	−
*δ*_2_ (model 6)	CWD × time trend squared	−	−	−	−	−	2.36E−6 * (1.35E−6)
*θ*_1_	Time trend	1.6500 *** (0.1405)	1.6153 *** (0.1502)	1.4459 *** (0.1252)	2.6818 *** (0.2878)	0.0313 *** (0.0034)	2.1598 *** (0.3451)
*θ*_2_	Time trend squared	−0.0004 *** (0.00003)	−0.0004 *** (0.00004)	−0.0004 *** (0.00003)	−0.0007 *** (0.00007)	−	−0.0005 *** (0.0001)
Obs.		50	50	50	50	50	50
*R* ^2^		0.9638	0.9653	0.9426	0.9634	0.9253	0.9668

^1^ Robust standard errors are in parentheses below each estimate. Superscripts *, **, and *** denote that the estimate is statistically significant at the 10%, 5%, and 1% level, respectively. ^2^ See Equation (1).

## Supplementary Materials

The dataset and Stata *.do files necessary to replicate our results are available online at https://www.mdpi.com/2076-2615/9/12/1096/s1.
