# Supplementary material for: The Effect of Chronic Wasting Disease on Resident Deer Hunting Permit Demand in Wisconsin
Source: Animals (Basel). 2019 Dec 7;9(12):1096. doi: 10.3390/ani9121096 (PMC6941111; doi:10.3390/ani9121096)
Supplement: Supplementary file 1 [file animals-09-01096-s001.zip › Supplemental Material.docx]

Supplemental Material:

The Effect of Chronic Wasting Disease on Resident Deer Hunting Permit Demand in Wisconsin

# S1. Comparison of the Distribution of Hunter Income with Wisconsin Resident Income

The US Fish & Wildlife Service [1] reports the distribution of Wisconsin resident income alongside the distribution of income of survey respondents who self-identify as hunters. Tables S1 and S2 reproduce this data for the years 2006 and 2001. Note that these reports present the most recent and complete estimates of hunter-level income. A test of proportions reveals no statistically significant difference between the shares of hunters and residents at each income level except for those in making between $40,000 and $49,999 yr^–1^ (2001 only) and $50,000 and $74,000 yr^–1^ (both years); there is a larger proportion of hunters in this income range than the general population. These findings support our use of mean Wisconsin resident income in estimating the demand for deer hunting permits.

## References

1. U.S. Census Bureau. Publications. https://www.census.gov/programs-surveys/fhwar/library/publications.html (accessed on 28 November 2019).

Table S1. Comparison of the Distributions of Wisconsin Resident and Wisconsin Hunter Incomes, 2006^1^

|  | Percentage | |  | Number | |  |  |  |
| --- | --- | --- | --- | --- | --- | --- | --- | --- |
| Income | General population | Hunters^2^ |  | General population | Hunters |  | Test statistic | *p*-value |
| < $10,000 | 3 | – |  | 119 | – |  | – | – |
| $10,000 to $19,999 | 5 | – |  | 214 | – |  | – | – |
| $20,000 to $29,999 | 10 | – |  | 415 | – |  | – | – |
| $30,000 to $39,000 | 11 | 15 |  | 492 | 101 |  | 1.17 | 0.12 |
| $40,000 to $49,999 | 8 | 11 |  | 328 | 71 |  | 0.84 | 0.20 |
| $50,000 to $74,999 | 21 | 27 |  | 894 | 178 |  | 1.79 | 0.04 |
| $75,000 to $99,999 | 8 | 12 |  | 350 | 81 |  | 1.2 | 0.12 |
| >$100,000 | 13 | 16 |  | 562 | 104 |  | 0.84 | 0.20 |

^1^ Source: [1].

^2^ Some data are missing due to an insufficient sample size; see [1].

Table S2. Comparison of the Distributions of Wisconsin Resident and Wisconsin Hunter Incomes, 2001^1^

|  | Percentage | |  | Number | |  |  |  |
| --- | --- | --- | --- | --- | --- | --- | --- | --- |
| Income | General population | Hunters^2^ |  | General population | Hunters |  | Test statistic | *p*-value |
| < $10,000 | 4 | – |  | 150 | – |  | – | – |
| $10,000 to $19,999 | 5 | – |  | 205 | – |  | – | – |
| $20,000 to $29,999 | 11 | 8 |  | 465 | 45 |  | -0.6 | 0.27 |
| $30,000 to $39,000 | 11 | 13 |  | 451 | 77 |  | 0.52 | 0.30 |
| $40,000 to $49,999 | 11 | 16 |  | 464 | 94 |  | 1.41 | 0.08 |
| $50,000 to $74,999 | 18 | 27 |  | 731 | 160 |  | 2.68 | 0.00 |
| $75,000 to $99,999 | 11 | 11 |  | 428 | 67 |  | 0 | 0.50 |
| >$100,000 | 7 | 7 |  | 285 | 42 |  | 0 | 0.50 |

^1^ Source: US FWS 2006.

^2^ Some data are missing due to an insufficient sample size; see [1].
